# Image based Machine Learning for identification of macrophage subsets

**DOI:** 10.1038/s41598-017-03780-z

**Published:** 2017-06-14

**Authors:** Hassan M. Rostam, Paul M. Reynolds, Morgan R. Alexander, Nikolaj Gadegaard, Amir M. Ghaemmaghami

**Affiliations:** 1 Division of Immunology, School of Life Sciences, Faculty of Medicine and Health Sciences, University of Nottingham, Nottingham, NG7 2RD UK; 2 Division of Biomedical Engineering, School of Engineering, University of Glasgow, Glasgow, G12 8LT UK; 3 Advanced Materials and Healthcare Technologies Division, School of Pharmacy, University of Nottingham, Nottingham, NG7 2RD UK; 4 Department of Biology, University of Garmian, Kalar, Kurdistan Iraq

## Abstract

Macrophages play a crucial rule in orchestrating immune responses against pathogens and foreign materials. Macrophages have remarkable plasticity in response to environmental cues and are able to acquire a spectrum of activation status, best exemplified by pro-inflammatory (M1) and anti-inflammatory (M2) phenotypes at the two ends of the spectrum. Characterisation of M1 and M2 subsets is usually carried out by quantification of multiple cell surface markers, transcription factors and cytokine profiles. These approaches are time-consuming, require large numbers of cells and are resource intensive. In this study, we used machine learning algorithms to develop a simple and fast imaging-based approach that enables automated identification of different macrophage functional phenotypes using their cell size and morphology. Fluorescent microscopy was used to assess cell morphology of different cell types which were stained for nucleus and actin distribution using DAPI and phalloidin respectively. By only analysing their morphology we were able to identify M1 and M2 phenotypes effectively and could distinguish them from naïve macrophages and monocytes with an average accuracy of 90%. Thus we suggest high-content and automated image analysis can be used for fast phenotyping of functionally diverse cell populations with reasonable accuracy and without the need for using multiple markers.

## Introduction

As a component of the innate immune system, macrophages play a central role in defence against pathogens as well as maintaining the body’s haemostasis. They achieve these by contributing to a number of functions including clearance of dead cells and microorganisms, recruitment of other immune cells and acting as antigen presenting cells (APCs) where they are able to provide necessary signals for T cell activation^1–3^. Different macrophage phenotypes with distinct functional properties have been identified^4^. For instance, M1 (classically activated) macrophages are induced by interferon gamma (IFN-γ) from T helper 1 (T_H_1) cells, CD8^+^ cytotoxic T cells (CTLs) or natural killer (NK) cells in the presence of microbial products such as lipopolysaccharide (LPS)^5^. M1 macrophages have pro-inflammatory and anti-tumour functions^4^ and secrete high levels of pro-inflammatory cytokines such as interleukin 12 (IL-12) and IL-23^6^. On the other hand, M2 (alternatively activated) macrophages are induced by IL-4 and/or IL-13, which are mainly secreted by T_H_2 cells^5^ or polymorphonuclear cells such as mast cells^7^. M2 macrophages have anti-inflammatory and pro-wound healing activities^4^ and secrete large amounts of the anti-inflammatory cytokine IL-10^8^.

*In vitro*, monocytes can be polarised towards M1 phenotype by IFN-γ^9^ or LPS^10^. The addition of granulocyte macrophage colony-stimulating factor (GM-CSF, which acts as a priming signal for macrophages^11, 12^ during M1 polarisation) augments the pro-inflammatory function of these cells^9, 13^. By contrast, M2 polarisation can be achieved by the addition of IL-4^9^. As with GM-CSF and M1 polarisation, macrophage colony-stimulating factor (M-CSF) can enhance the anti-inflammatory function of M2 macrophages^9, 13^.

Human macrophages express the intracellular marker CD68 and this is often used to identify them in tissue samples^14^. In order to determine the activation status of macrophages, typically a panel of surface markers, cytokines, metabolites or transcription factors are employed. For instance, M1 macrophages can be identified by the production of high levels of pro-inflammatory cytokines such as IL-12, IL-23^6^, IL-1β, IL-6 and tumour necrosis factor alpha (TNF-α)^15, 16^. These cells have also been shown to express high levels of chemokine (C-C motif) receptor 7 (CCR7)^17^, nitric oxide synthase 2 (NOS2)^18^, calprotectin^19^, and CCR2^20^. Conversely, M2 macrophages are characterised by the production of high levels of IL-10^21^, transforming growth factor beta (TGF-β)^16^, and high expression of the scavenger receptor CD163^18, 21^, mannose receptor (MR, CD206)^17, 21^ and IL-1 receptor antagonist (IL-1RA)^22^.

In terms of gene expression and transcription factor activation, the main characteristics of human M1 macrophages are high levels of prostaglandin-endoperoxide synthase 2 (Ptgs2 or Cox2) and IL23a (IL23p19) gene expression, and signal transducer and activator of transcription 3 (STAT3) and/or STAT1 phosphorylation. In addition, high levels of SOCS3 and IRF5 expression have also been associated with pro-inflammatory M1 macrophages *in vitro* ^23–26^. Human M2 macrophages, on the other hand, can be identified by high levels of Kruppel-like factor 4 (Klf4) and chitinase 3-like 2 (Chi3l2 or Ykl39) gene expression and STAT6 phosphorylation^27^.

Unlike in murine macrophages where M1 and M2 activation results in distinct marker profiles, in human macrophages there is overlap in the expression of markers between the two activation states. For example, Arginase-1 (Arg1), which has been used as an “M2 marker” in murine macrophages, has been detected in both M1 and M2 human macrophages^28^. In addition, MR and chemokine (C-C motif) ligand 18 (CCL18) (M2 markers) can also be expressed by M1 like macrophages^29^. Thus, it appears that in human macrophages, differences between marker expression in M1 and M2 phenotypes are more quantitative rather than qualitative^30^.

Given the complexity of M1/M2 identification in human macrophages, we sought an alternative approach that would be simpler, less resource-intensive and hence more widely adoptable. Studies that focussed on the morphology of different macrophage phenotypes^29, 31–36^ led us to hypothesise that cell morphology could be an indicator of macrophage activation status.

Pelegrin and Surprenant reported that murine peritoneal macrophages polarised *in vitro* to an M1 phenotype were distended cells with multiple lamellar processes, elongated filopodia, and distributed F-actin in the cytoplasm. On the other hand, *in vitro* polarisation of these macrophages to an M2 phenotype resulted in cells that were similar in shape to unpolarised macrophages, with less lamellar processes and paranuclear-compacted F-actin^33^. Vereyken *et al*. also observed a relationship between macrophage activation and morphology in murine bone marrow-derived macrophages. Their data suggested that M1 macrophages appeared large, rounded and flat while M2 macrophages were stretched and elongated cells^36^. Furthermore, a recent study by McWhorter *et al*. succeeded in polarising murine bone marrow-derived macrophages towards an M2 phenotype by inducing the cells to adopt an elongated morphology on micropatterned grooves with a width of 20 μm^35^. These data suggest that not only different macrophage phenotypes have distinct morphologies but also altering the morphology of macrophages can itself trigger alterations in the activation status of these cells.

Morphological differences have also been seen in differentially activated human macrophages. Using the human monocyte-like cell line THP-1, Lee *et al*. observed a correlation between cell morphology and the production of the pro-inflammatory cytokine TNF-α^34^. Porcheray *et al*. also found that different pro- and anti-inflammatory cytokines induced distinct morphologies in human primary monocyte-derived macrophages, although in their study, there was no correlation between macrophage morphology and surface expression of a selection of M1 (e.g. MIP-1α) and M2 (e.g. MR, CD163, CCL18) markers, highlighting the limitations of relying on surface markers for determining macrophage phenotype^29^.

The aim of the present study was to further investigate the relationship between morphology and activation status in human primary monocyte-derived macrophages. Monocytes were stimulated *in vitro* with M1 or M2 inducing cytokines for 6 days following which macrophage phenotype was confirmed by immunofluorescent staining for calprotectin and MR expression (M1 and M2 surface markers respectively^37^), measurement of cytokines in culture supernatants, and analysis of transcription factors by quantitative real-time PCR (qRT-PCR). Unpolarised (naïve) macrophages, freshly isolated monocytes, and monocytes cultured for 6 days without cytokines were also included as controls. Macrophage morphology was assessed microscopically by staining the cells with fluorescently labelled phalloidin in order to visualise the actin cytoskeleton. Cell images were analysed using CellProfiler^38, 39^ in order to measure different dimensions of the cells and their nuclei, and create a specific profile with shared characteristics for each cell type. These profiles formed the basis for M1 and M2 phenotype identification. From the automated CellProfiler analysis a so-called ‘cytoprofile’ is established which describes the characteristics of the individual cells from different phenotypes. This profile includes size, shape, intensity, and texture of the actin and nuclear stain. With the cytoprofile established for the different cell types, this large multivariate dataset was used to create a classifier based on various machine learning algorithms. The Orange data mining toolbox provides a graphical user interface, allowing users to visually build data flows, train classifiers, and score predictions on this type of multivariate data. Beyond simply visualising the cell body, fluorescent labelling of the nucleus and actin cytoskeleton provides a wealth of information^38^. Common descriptors of cell morphology such as cell area, perimeter, and elongation can be combined with more specific metrics of texture and intensity to create a robust ‘fingerprint’ of a given phenotype – referred to as the ‘cytoprofile’. A number of open source packages have been released in recent years which allow researchers to utilise this ‘cytoprofile’ to perform multivariate and machine learning analyses^38, 40^. In this study we used a number of supervised classifiers^39^ available in Orangedata mining toolbox^41^ to construct a 5-way classifier capable of distinguishing between monocyte and different macrophage phenotypes.

Machine learning has found numerous applications in biology in recent years^42–44^, from RNA screening studies detecting over 50 phenotypes^39^ down to the simple classification of two cell types from a population (48). Methods applying such high content image analysis to the detection and classification of various cell types have been demonstrated, including in mesenchymal stem cells^45^ and endothelial/fibroblast cells^46^. Beyond this, machine learning methods have also been used to classify the specific stage of the cell cycle, e.g. M-phase^47^, and to assist clinicians in diagnostic settings^44^. In this study, we propose the use of a supervised classifier to distinguish between M1 and M2 macrophages. Machine learning effectively substitutes the manual classification of cell phenotype by researchers and clinical practitioners and offloads the decision making to an algorithm. The reasons automated image processing, segmentation and analysis have found such interest are that they offer huge possibilities to free up researchers time and to ensure consistent interpretation of results free from any human bias.

## Material and Methods

### Monocyte Isolation

Buffy coats form healthy donors were obtained from the National Blood Service (National Blood Service, Sheffield, UK) following Ethics committee approval (2009/D055). Peripheral blood mononuclear cells (PBMCs) were isolated from heparinised blood by Histopaque-1077 (Sigma-Aldrich) density gradient centrifugation. Monocytes were isolated from PBMCs using the MACS magnetic cell separation system (positive selection with CD14 MicroBeads and LS columns, Miltenyi Biotec) as described before^48, 49^. This method routinely yielded >95% pure monocytes as determined by flow cytometric analysis of CD14 expression. All methods were performed in accordance with the relevant guidelines and regulations.

### Macrophage culture and activation

Purified monocytes were suspended in RPMI-1640 medium supplemented with 10% foetal bovine serum (FBS), 2 mM L-glutamine, 100 U/ml penicillin, and 100 μg/ml streptomycin (all from Sigma-Aldrich) (henceforth referred to as “complete RPMI medium”) and seeded at 1 × 10^6^ cells/ml/well in 24-well tissue culture-treated polystyrene (TCP) plates (Corning Life Sciences). The following cytokines were added to the monocytes to obtain different activation states as we have described before^37^: M1–20 ng/ml IFN-γ (R&D Systems) and 50 ng/ml GM-CSF (Miltenyi Biotec); M2–20 ng/ml of IL-4 (Miltenyi Biotec) and 50 ng/ml M-CSF (Miltenyi Biotec)^50^; naïve (unpolarised) macrophages – 50 ng/ml GM-CSF. Monocytes not treated with any cytokines served as untreated controls. The cells were incubated at 37 °C, 5% CO_2_ in a humidified incubator for 6 days. On Day 3 of incubation, 500 μl of the medium in the wells was replaced with fresh complete RPMI medium containing the same concentration and mix of cytokines that were used for cell stimulation on Day 0. Cell supernatants and mRNA were harvested on Day 6 for cytokine analysis and qRT-PCR, respectively.

For microscopy, round coverslips with 12 mm diameter, thickness #2 (VWR International) coated with 20 μg/ml Poly-L-lysine hydrobromide (Sigma-Aldrich)^51^ were put inside each well of a 24-well TCP plate. Monocytes were then seeded and stimulated on the coverslips as described above. On Day 6 cells were washed once with PBS and fixed as per the protocol below.

### Fluorescence microscopy

#### Immunocytochemistry for calprotectin, MR and CD68

On day 6, adherent cells on coverslips were fixed with 4% paraformaldehyde (EMS Diasum) in PBS for 10 min. Fixation and all subsequent steps in this procedure were carried out at room temperature; all washes were carried out with 0.2% Tween 10 (Sigma-Aldrich) in PBS (5 min per wash) except where stated. Following fixation, cells were washed twice, then blocked with 1% (*w*/*v*) glycine (Fisher Scientific) and 3% (*v*/*v*) bovine serum albumin (BSA, Sigma-Aldrich) in PBS for 30 min. Subsequently, cells were washed twice and incubated for 30 min with 5% (*v*/*v*) goat serum (Sigma-Aldrich) in PBS to block non-specific antibody binding. Next, cells were incubated for 1 h with the appropriate primary antibody (see Table 1), washed 3 times, and then incubated for 1 h with the appropriate secondary antibody at room temperature (see Table 1). Finally, all cells were stained with 250 ng/ml DAPI (4′,6-Diamidino-2-Phenylindole) (Life Technologies) in PBS for 5 min, washed 3 times with PBS, covered with fluorescent mounting medium (Sigma-Aldrich) and mounted on slides. Samples were imaged using a Leica DMRB florescence microscope with monochrome digital camera (Hammamatsu C4742-95) and OpenLab software.

**Table 1:** Primary and secondary antibodies used for immunocytochemistry.

	All cells (CD68)	M1-induced cells (Calprotectin)	M2-induced cells (MR)
**Primary antibody**	Mouse monoclonal anti-human CD68	Mouse monoclonal anti-human calprotectin	Rabbit polyclonal anti-human MR
Clone	EBM11	27E10	ab64693
Source	DakoCytomation	Thermo Scientific	Abcam
Concentration	4.7 µg/ml	2 µg/ml	1 µg/ml
**Secondary antibody**	Goat anti-mouse IgG (H + L)	Goat anti-mouse IgG (H + L)	Goat anti-rabbit IgG (H + L)
Conjugate	Alexa Fluor 488	Rhodamine red	Alexa Fluor 488
Source	Life Technologies	Life Technologies	Life Technologies
Concentration	8 µg/ml	8 µg/ml	8 µg/ml

#### Morphological analysis of different cell types

Cells were fixed with 4% paraformaldehyde in PBS as described above, washed twice with PBS (5 min per wash), then permeabilized by 0.2% Triton-X100 (Sigma-Aldrich) in PBS for 20 min. All the steps in this procedure were carried out at room temperature. After 2 washes with PBS, non-specific binding was blocked with 5% goat serum in PBS as described in the previous section. This was followed by 2 washes with PBS and cytoskeleton staining of F-actin with 5 μg/ml Alexa Fluor® 488 Phalloidin (Cell Signalling Technology) in 1% goat serum and 0.1% sodium azide (Sigma-Aldrich) for 30 min. Cells were then washed 3 times with PBS and stained with 250 ng/ml DAPI (4′,6-Diamidino-2-Phenylindole) (Invitrogen) in PBS for 5 min, washed 3 times with PBS, then embedded with anti-fade medium, and finally mounted on a slide followed by imaging using a florescent microscope (LEICA).

#### Cytokine analysis

TNF-α, IL-6, IL-8, and IL-1β production was assessed by means of the FlowCytomix bead-based multiplex system (eBioscience) as described before^52–54^. Briefly, 10 μl of sample or standard was mixed with 10 μl of a cocktail of beads coated with primary antibodies for the detection of the cytokines of interest and 20 μl of a cocktail of biotin-conjugated secondary antibodies in a FACS tube and incubated for 2 h at room temperature in the dark. Tubes were then washed twice with 400 μl each of assay buffer (provided in the kit) to removed unbound beads and antibodies. 20 μl of diluted streptavidin-phycoerythrin (PE) conjugate was then added to each tube and the samples/standards incubated for 1 h at room temperature in the dark. Samples were washed twice with assay buffer as before and resuspended in 400 μl assay buffer, stored at 4 °C, and analysed on a Beckman Coulter FC500 flow cytometer within 24 h. Results were analysed using the eBioscience FlowCytomix Pro 3.0 software.

CCL18 production was measured using the Human CCL18/PARC DuoSet ELISA kit (R&D Systems) as per the manufacturer’s instructions.

IL-1RA and IL-10 production were measured using the ProcartaPlex bead-based luminex system (Affymetrix eBioscience) as per the manufacturer’s instructions. Plates were read on a Bio-Rad Bio-Plex 200 system and results analysed using the ProcartaPlex Analyst 1.0 software.

#### RNA extraction, cDNA conversion and qRT-PCR

Total RNA was extracted from cells using the RNeasy Plus Minikit (Qiagen) according to the manufacturer’s protocol. The concentration of the total RNA was determined by Nanodrop ND1000 spectrophotometer (Thermo scientific) according to the manufacturer’s protocol. cDNA was synthesized from 1 µg of total RNA using superscript III first-strand synthesis kit (Invitrogen). qRT-PCR was performed based on TaqMan and SYBR chemistry using LightCycler® 96 machine (Roche) using the following primer sequences (Table 2). The data were analysed by LightCycler® 96 SW 1.1 software (Roche).

**Table 2:** Primer sequences used in rRT-PCR experiments.

Genes	Primers/probe	Sequence (5′-3′)
STAT1	Forward	GGAAGGGGCCATCACATTCA
	Reverse	GTAGGGTTCAACCGCATGGA
STAT6	Forward	CTCGCTGGACAGAGCTACAG
	Reverse	CCCTCTGCTGTCTTCTCCCT
SOCS1	Forward	CCCTGGTTGTTGTAGCAGCTT
	Reverse	TTGTGCAAAGATACTGGGTATATGT
IRF5	Forward	GCCATGAGCAGGGAAAGAAC
	Reverse	CCCTTAGGCAATTCCTCCTATACA
SOCS3	Life Technologies Hs02330328_s1 (*Taqman*)	
IRF4	Life Technologies Hs01056533_m1 (*Taqman*)	

For the normalization, glyceraldehyde 3-phosphate dehydrogenase (*GAPDH*) was chosen as the house-keeping gene. Relative expression of each gene of interest was calculated based on the relative standard curve method. The relative expression of the target gene was determined by the concentration of target gene divided by the concentration of *GAPDH*.

#### Image analysis and machine learning for phenotype identification

Composite images of f-actin and DAPI staining were loaded into CellProfiler and metadata detailing the cell type in the image was extracted from the file name. After optimisation of the primary detection of the cell nucleus (DAPI channel), followed by secondary detection of the cell body (f-actin channel) the full dataset of 93 images was analysed in 4 h on a high spec desktop computer (Dell XPS). Each cell was then morphologically analysed for a broad range of descriptors such as area, orientation, extent, shape, intensity, etc. A total of 228 measurements were acquired for each cell, and a database was established for the full experiment, which was comprised of 150 images. The machine learning step was then carried out in Orange Data Mining Toolbox. Here, workflows were created to import and format the morphometric data, using cell types as class identifiers. There were 5 cell types assessed in this study along with a set of 40 blind images not seen by the classifier. After data import, five classification methods were set up with operational parameters chosen based on previous experience. A random subsection of the data was used to train each classifier, followed by testing classification accuracy on the remaining data. Classification accuracy was measured by 10-fold cross validation, after which minor modifications were made to classifier parameters to improve performance. The parameters used in the Orange toolbox are listed in the supplementary section.

#### Statistical analysis

Statistical significance of differences between different expression profiles was assessed using Student’s t-test with GraphPad Prism 6 Differences were considered statistically significant if the p-value was less than 0.05.

## Results

### Characterisation of macrophage activation status

Human peripheral blood monocytes were differentiated into macrophages *in vitro* in the presence of GM-CSF (naïve macrophages), IFN-γ and GM-CSF (M1 macrophages) or IL-4 and M-CSF (M2 macrophages) for 6 days. In order to establish the differentiation status of these monocyte-derived macrophages using traditional methods, immunofluorescent staining for the M1 and M2 markers, calprotectin and MR respectively, was performed. Untreated monocytes cultured *in vitro* for 6 days were used as controls. In addition, further characterisation of M1 and M2 macrophages was carried out by analysing the cytokine profiles of these cells after 6 days of culture and determining their transcription factor expression profile by qRT-PCR.

**Figure 1:**
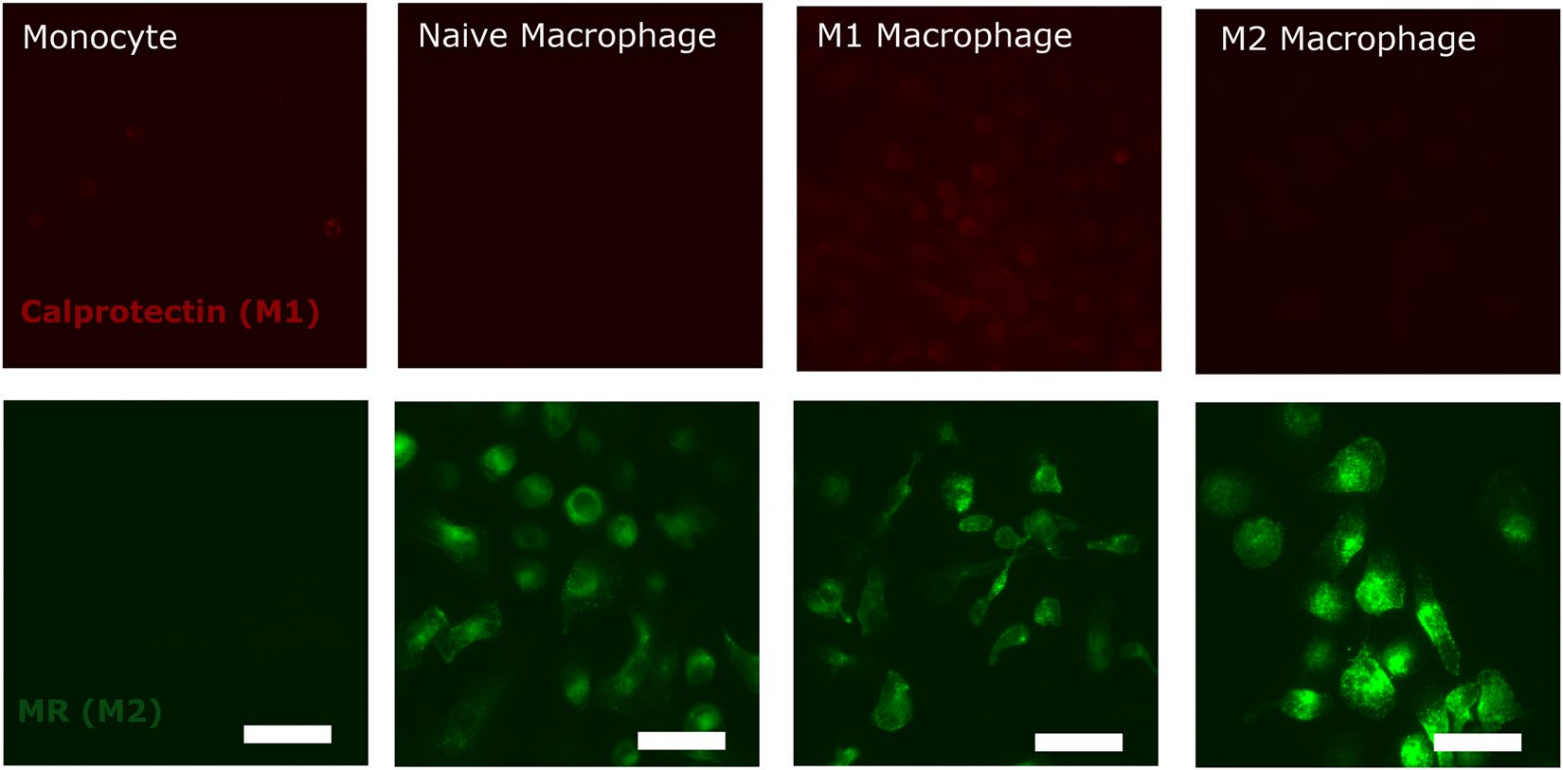
Fluorescent images of monocytes and macrophages stained for calprotectin (27E10 antigen, red, **A**–**D**), and mannose receptor (MR, green, **E**–**H**). Scale bar = 25 µm. Representative images from 6 independent biological samples (donors) are shown.

Immunofluorescent staining for the activation markers calprotectin and MR (CD206) demonstrated that M1 macrophages had the highest expression of calprotectin, while expression of this marker was much lower on M2 macrophages and naïve macrophages and not detected in untreated monocytes (Fig. 1). On the other hand, expression of MR was found to be highest on M2 macrophages followed by M1 macrophages, naïve macrophages and untreated monocytes in that order (Fig. 1). CD68, a macrophage marker that was included in order to determine macrophage differentiation, was expressed in all the macrophage types after 6 days in culture, but not in untreated monocytes (Supplementary section).

**Figure 2:**
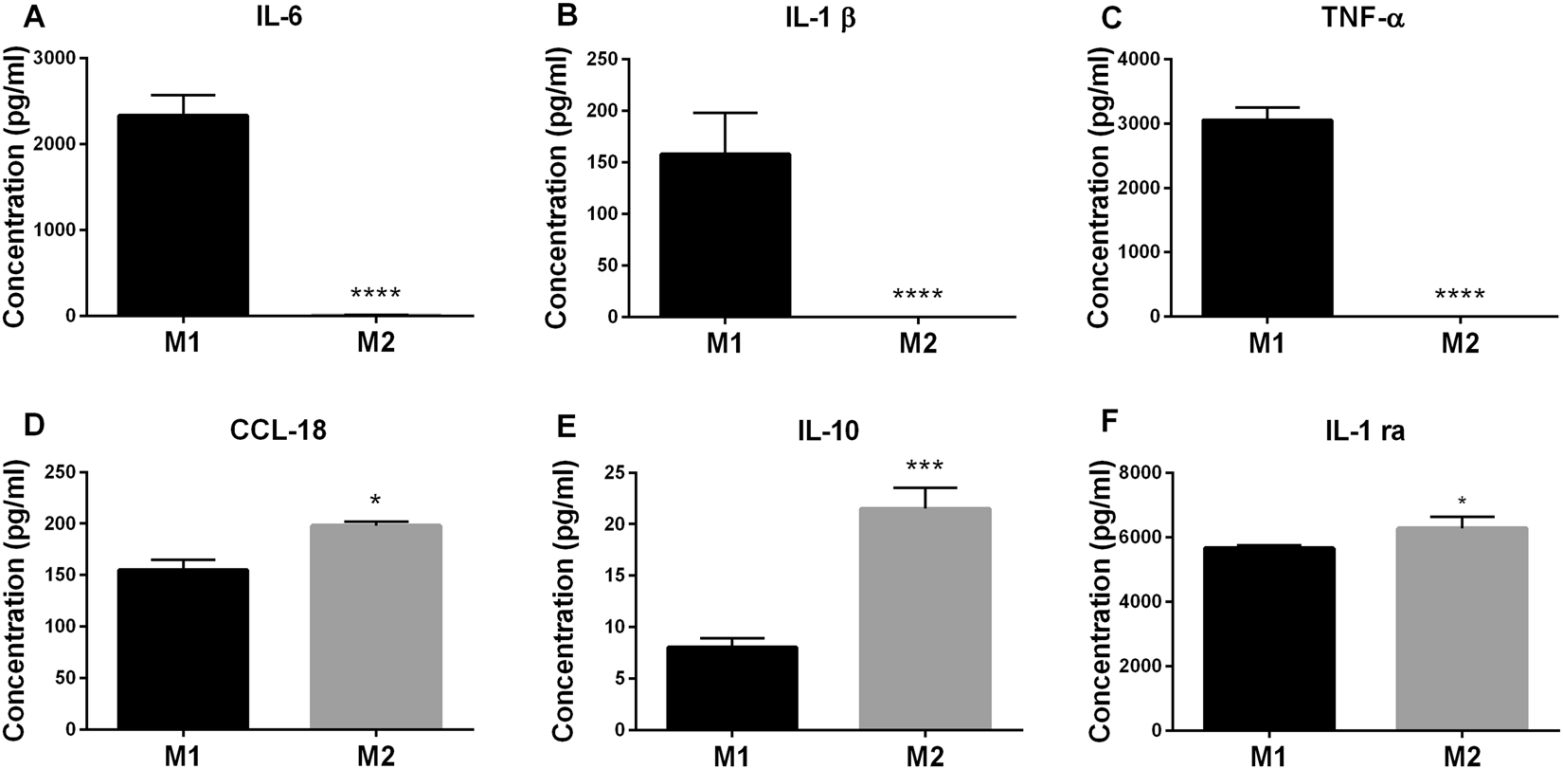
Comparison of cytokine profiles of M1 and M2 macrophages. Cytokines in the supernatants of human monocyte-derived M1 and M2 macrophages cultured for 6 days were measured by means of a bead-based flow cytometric system (for (**A**) IL-6, (**B**) IL-1β, (**C**) TNF-α), ELISA (for (**D**) CCL-18), and a bead-based luminex system (for (**E**) IL-10 and (**F**) IL-1RA). Data presented are the mean ± SD of at least 5 independent experiments using blood samples from different donors. Statistical significance was assessed using paired Student’s t-test (*P < 0.05, **p < 0.01, ***p < 0.001).

Cytokine analysis of supernatants obtained from M1 and M2 macrophages after 6 days of culture demonstrated that M1 macrophages produced significantly higher amounts of the pro-inflammatory cytokines IL-6 (p < 0.000)1, IL-1β (p < 0.0001), and TNF-α (p < 0.0001) (Fig. 2). By comparison, M2 macrophages produced significantly higher levels of the cytokines IL-10 and IL-1RA (p = 0.0115), and the chemokine CCL18 (p = 0.0245) (Fig. 2), which play a role in the reduction of inflammation and wound-healing.

**Figure 3:**
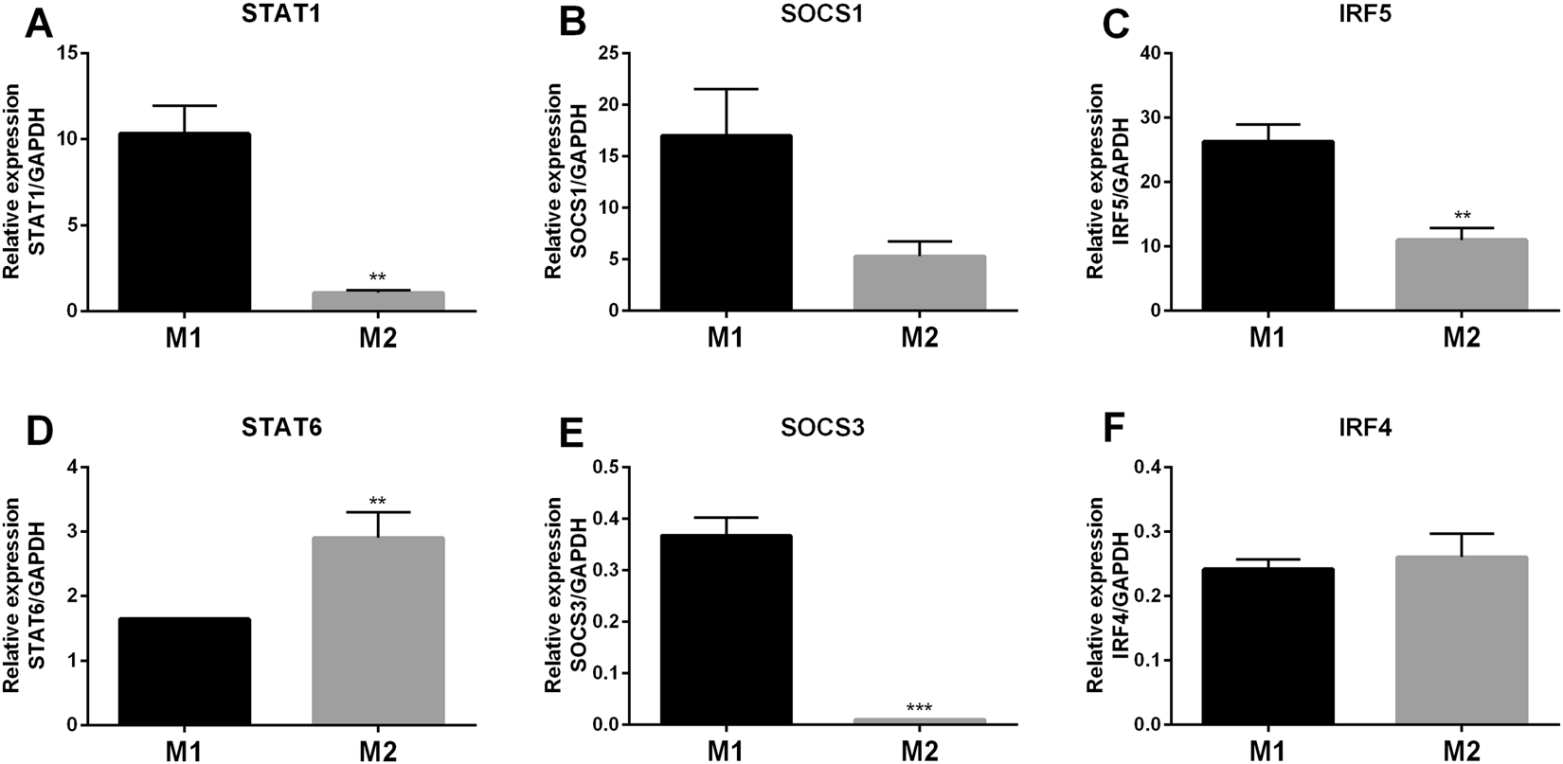
Comparison of transcription factor mRNA expression in M1 and M2 macrophages. qRT-PCR analysis of (**A**) STAT1, (**B**) SOCS1, (**C**) IRF5, (**D**) STAT6, (**E**) SOCS3, (**F**) IRF4 relative mRNA expression in M1 and M2 macrophages after 6 days of culture. All values are reported relative to the house-keeping gene GAPDH. Data show mean values ± SEM of 3 independent experiments using macrophages generated from 3 different donors. Statistical significance was assessed using student’s t-test (**p < 0.01, ***p < 0.001).

To further characterise the phenotype of polarised macrophages, qRT-PCR was used to determine the relative mRNA expression of a panel of transcription factors. There was significantly higher expression of STAT1 (p < 0.0050) (Fig. 3A), suppressor of cytokine signalling 3 (SOCS3 (p < 0.0005), Fig. 3E), and interferon regulatory factor (IRF5 (p < 0.0055), Fig. 3C) mRNA in M1 macrophages in comparison with M2 macrophages. SOCS1 mRNA expression was also higher in M1 compared to M2 macrophages however this was not statistically significant (Fig. 3B). M2 macrophages, on the other hand, expressed significantly more STAT6 (p < 0.0053) mRNA than M1 macrophages (Fig. 3D). A small, non-significant increase in IRF4 mRNA was also noted in M2 macrophages compared to M1 macrophages (Fig. 3F).

### Characterisation of Macrophage Morphology

**Figure 4:**
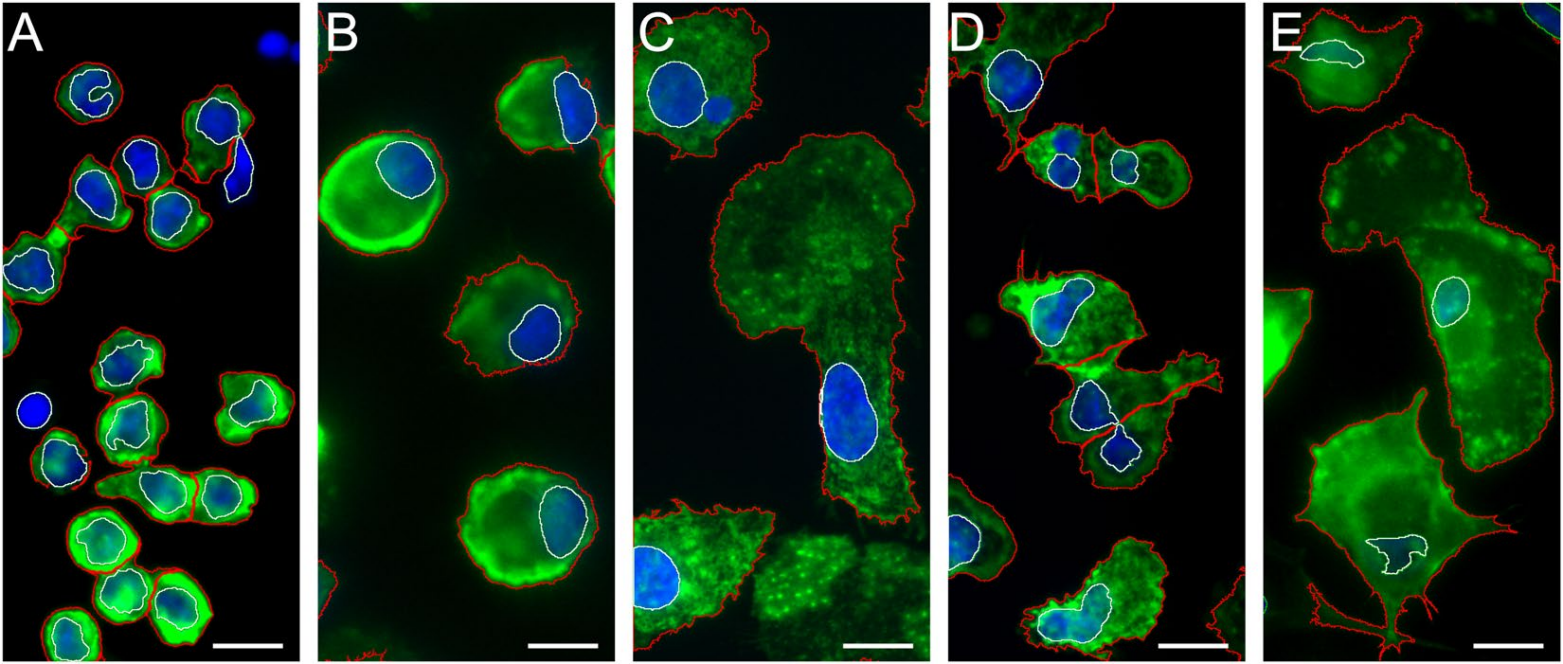
Immunofluorescent staining of monocytes and macrophages. (**A**) Monocytes cultured for 2 h; (**B**) monocytes cultured for 6 days; (**C**) naïve macrophages; (**D**) M1 macrophages; (**E**) M2 macrophages. F-actin and cell nuclei were stained with Phalloidin Alexa Fluor 488 (green) and DAPI (blue), respectively. The detected nucleus and cell outlines are shown as green and red lines respectively. Scale bar = 10 µm. Representative images from n = 6 are presented.

In order to visualise the morphology of naïve, M1 and M2 macrophages, cells were stained for their nuclei and f-actin filaments using DAPI and fluorescently labelled phalloidin, respectively. Untreated monocytes cultured for 2 h and 6 days were used as controls. Morphological differences were observed between the different cell types. Monocytes cultured for 2 h were found to be small rounded cells (Fig. 4A). After 6 days in culture, monocytes appeared as larger rounded cells, whilst naïve macrophages were larger and more irregular in shape (Fig. 4B and C, respectively). M2 macrophages were the largest of the five cell types, showing a flattened, expanded phenotype (Fig. 4E). M1 macrophages, on the other hand, were smaller, irregular-shaped cells, some of which adopted an elongated spindle-shaped appearance (Fig. 4D). These images were analysed to obtain different parameters (e.g. nuclei to cytoplasm ratio, cell perimeter) for each cell phenotype which were processed in the CellProfiler software suite (Broad Institute, Harvard, USA). A description of the way in which a number of these measurements are calculated is included in the supplementary section. These measurements can be generated using immunofluorescent data from many different cell types^46, 55^.

**Figure 5:**
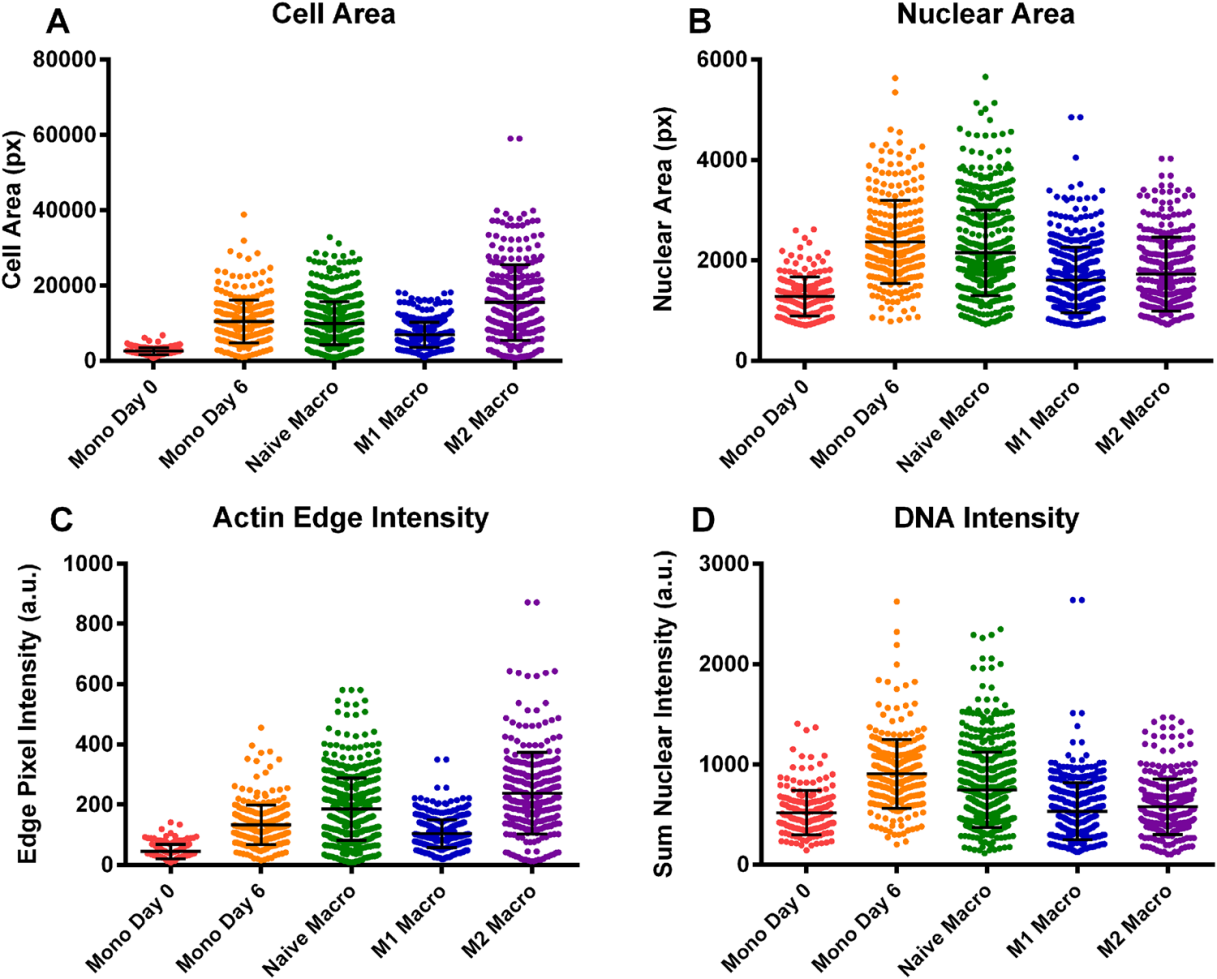
Comparison of key morphometric identifiers. Cell area (**A**), nuclear area (**B**), sum edge actin intensity normalised to cell area (**C**) and sum nuclear DNA intensity normalised to nuclear area (**D**) are shown as histograms for at least 500 cells of each type. The uniformity in size and shape is evident for monocytes observed at day 0, after which the characteristics of each cell type separate. M2 macrophages appear to have the largest cell area, whilst monocytes cultured for 6 days exhibit the largest nuclei. It follows that their nuclear DNA intensity is the highest. Actin edge intensity and cell area appear to correlate, suggesting a well-defined cytoskeleton in larger immune cells – more so in naïve macrophages than in monocytes.

Visual inspection of cytoskeletal staining of macrophages showed immediate differences in their respective morphologies. Beyond basic descriptors such as size and shape, there were differences in the distribution and texture of the cell cytoskeleton. These differences can be quantified and described in detail by various image processing methods^38^. Significant differences emerged between M1 and M2 macrophages. M1 macrophages were smaller, more rounded cells with tightly packed dotted texture of actin, Fig. 4D. M2 macrophages exhibited larger, more irregular cell bodies with smoother actin staining and more distributed localised spots, Fig. 4E. Various metrics of nucleus/cell size, texture, and staining intensity show differences in M1 and M2 phenotypes, as shown in Fig. 5 differences in cell size (Fig. 5A) and nuclear size (Fig. 5B) were confirmed, whilst other metrics emerged as key differences between the two phenotypes such as the intensity of actin staining around the cell periphery (Fig. 5C) and the sum intensity of nuclear DNA staining (Fig. 5D). The distribution of each metric varies across cell types – with some allowing clear separation and others showing little variation.

### Classification of Cell Type by Machine Learning

Alongside the ability of immunostaining, cytokine profile and PCR (Figs 1–3 respectively) to confirm M1/M2 phenotype we demonstrate that simple descriptors of cell shape also hold sufficient information to allow accurate identification of macrophage polarisation states. The inherent heterogeneity of the cell system requires that multiple nuances in cell shape within individual phenotypes can be identified and classified. Of the 228 measurements collected for each cell, some were found to significantly differ between phenotypes, whilst others did not (Fig. 5). The heterogeneity of each cell type is also apparent from the distribution of values presented in Fig. 5. Monocytes imaged after 2 h exhibited small, rounded morphologies in which the nucleus occupied the majority of the cell volume (Fig. 4A). Nevertheless we propose that there are unique feature sets contained within the cytoprofile of each cell which will allow accurate segregation using a supervised classifier. A supervised rather than unsupervised classifier is used in this instance as large datasets of pre-sorted cells are obtainable from primary sources – providing a robust training set from which to build a classifier.

**Figure 6:**
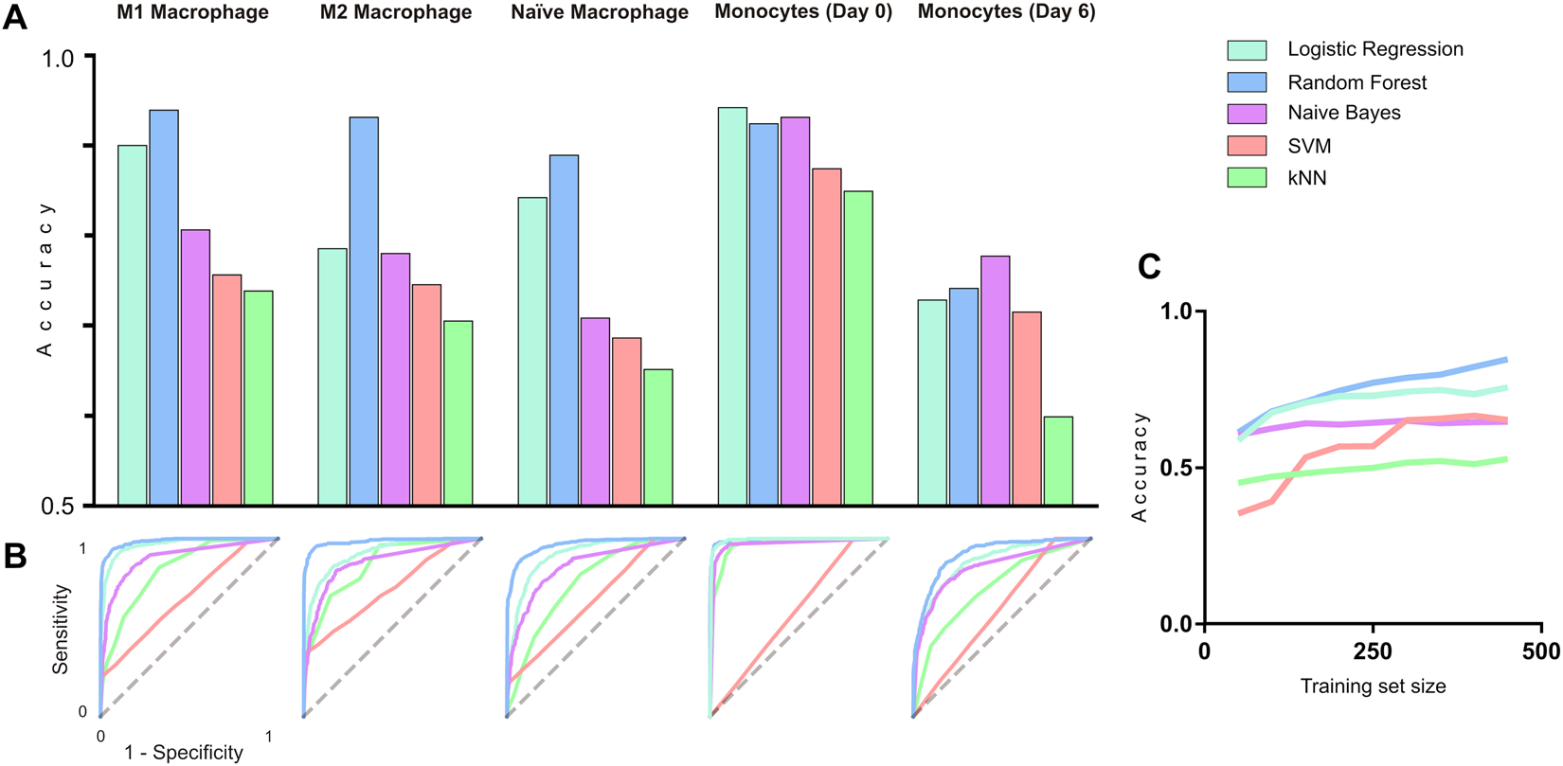
Comparing classifier accuracy in determining M1 and M2 phenotype. (**A**) Classification accuracy of 5 immune cell types using 5 classifiers assessed by 10-fold cross validation. Logistic regression and random forest classifiers showed the highest accuracy for all cell types whilst support vector machine (SVM) and k-nearest neighbours (kNN) frequently misclassified cells. (**B**) ROC curves are plotted for each cell type as classifier sensitivity versus 1-specificity show the variation in classifier performance for each cell type. Dashed grey lines indicate a random guess. (**C**) The influence of training set size on the performance each classifier – accuracy is presented as an average of 10-fold cross validation across each cell type.

Five supervised machine learning methods were considered to build classifiers for image based segmentation of immune cell data. Support vector machine (SVM), k-nearest neighbour (kNN), naïve Bayes, logistic regression, and a random forest classifier^56^. Using the Orange data mining toolbox^41^, the data was divided into a randomly selected training set and test data. Classifiers were trained using 50% of the available data and tested on the remaining 50%. Classifier accuracy was validated by 10-fold cross validation and analysis of Receiver Operating Characteristic (ROC) curves for each cell type, Fig. 6. These classifiers differ in the way multivariate data is used to determine cell type. Due to the heterogeneity of cell morphologies both within and between classes, we used five classifiers to test performance (Supplementary Table 2). Cell clusters such as mitotic cells, cell debris and cells atop one another represent outliers – a problem which kNN classifiers are more susceptible to whilst SVM classifiers can partially successfully adapt to, given a sufficiently large training set. Logistic Regression is an easily adaptable method which assumes a linearly separable problem – in this case we expect step variations in features such as cell size, shape, and actin content which can be combined to accurately distinguish cell class. Random Forest classification does not assume that features are linear, and therefore in the case of binary features is more capable of drawing determinations. Finally, a Naïve Bayes classifier assumes that features are independent – in the case of multiple immune cell phenotypes and a rich feature set, we can assume that some metrics may be independent, whilst others intrinsically linked such as cell size and total actin intensity. As a consequence of the properties of each classifier trialled here, we can also describe the properties of our multivariate data – whether these macrophage morphologies show distinct differences in individual features, or more nuanced changes across phenotypes.

To examine the ability of the classifiers to correctly identify different immune cell phenotypes, we report the accuracy of each classifier for each immune cell phenotype, Fig. 6A. The k-nearest neighbour (kNN) and support vector machine (SVM) classifiers performed poorly across all phenotypes with the exception of monocytes imaged after 2 h culture. These cells were included as a negative control in the dataset to challenge the classifier with a phenotype which was starkly different from the others. We found no overfitting of any classifiers which prevented the identification of these monocytes with less than 95% accuracy. The results of classification using the SVM classifier appear to vary linearly (Fig. 6B) which is typically indicative of random results. SVM may be particularly unsuited to this type of classification exercise, where classes may exhibit outlying phenotypes and the number of features which vary significantly are relatively sparse. As the SVM fits a hyperplane with maximised distance between classes, it may be easily skewed with low sample numbers and training set sizes. This is also apparent from its staggered increase in accuracy with training set size (Fig. 6C). However, the linear regression model is more effective which is likely due to its use of a probability distribution to model the input training set and making class determinations based on such training set. Nevertheless, both the linear regression and the random forest models were capable of correctly identifying M1 and M2 macrophages from mixed image sets with accuracies in excess of 90%. ROC curves for each cell type and classifier also confirm the performance of the random forest classifier, and show that the naïve macrophage and monocyte after 6 days presented phenotypes which were difficult to classify, Fig. 6B. These inaccuracies may stem from the heterogeneous cell populations in hand, as they are derived from primary sources. However, even with lower accuracies all classifiers tested outperformed a random classifier, Fig. 6B.

Of the classifiers trialled in this study, a random forest classifier with 20 trees was found to be the most accurate in determining immune cell phenotype. A confusion matrix shows the percentage of cells which were correctly classified for each phenotype, Table 3. The classifier exceeded 85% accuracy for M1, M2 and naïve macrophages. The most common misclassifications were of monocytes imaged after 6 days with naïve macrophages.Given the very similar nature of day 6 monocytes and naive macrophages, this observation is not very surprising. Nevertheless, this misclassification can be explained by the dominance of cell area as a discerning metric in the decision tree. Whilst other cell types showed distinct distributions of cell area, these two phenotypes exhibit a near identical distribution (Fig. 5A). The large number of decision trees and subsequent nodes involved in a random forest classifier makes interpretation and location of the error source difficult.

**Table 3:** Confusion matrix showing machine learning classification of immune cell phenotypes.

		M1 Macrophage	M2 Macrophage	Naïve Macrophage	Monocyte (Day 0)	Monocyte (Day 6)
Cell Type	M1 Macrophage	**92**.**4**	0.4	4.1	1.1	2.0
	M2 Macrophage	6.3	**89**.**0**	4.3	0.0	0.3
	Naïve Macrophage	3.0	2.8	**88**.**8**	0.4	5.1
	Monocyte (Day 0)	7.4	0.5	2.1	**90**.**0**	0.0
	Monocyte (Day 6)	10	6.8	26.8	1.8	**54**.**6**

Data is presented for all cells as a percentage of correctly classified cells. Approximately 500 observations were classified for each cell type across 152 images using a random forest classifier. The number of correctly classified cells is presented as a percentage, with true positives highlighted on the diagonal in red. Confusion matrices for all 5 classifier types used in this work are included in the supplementary section.

## Conclusions

Image based machine learning using a broad array of metrics of the cell nucleus and cytoskeleton is an effective means of classifying M1 and M2 macrophages in mixed populations. These results demonstrate that an ‘off the shelf’ random forest classifier is capable of achieving accuracies in excess of 89% in classifying M1 and M2 macrophages. We have confirmed the phenotypical difference between Naïve, M1 and M2 macrophages isolated from peripheral blood by classical techniques as found in the literature, namely qRT-PCR, cytokine profiling and immunostaining. In addition, we have demonstrated a new means of classifying macrophage populations using image based machine learning. Given the heterogeneity of macrophage phenotype and current limitations of the machine learning approach it may be too early to suggest use of image analysis as an alternative to conventional cell phenotyping. However, our data provide strong evidence for the ability of high content and automated image analysis approaches for accurate, less resource intensive and fast phenotyping of functional diverse cell populations.

## Electronic supplementary material

Supplementary Information
