# Supplementary material for: Image based Machine Learning for identification of macrophage subsets
Source: Sci Rep. 2017 Jun 14;7:3521. doi: 10.1038/s41598-017-03780-z (PMC5471192; doi:10.1038/s41598-017-03780-z)
Supplement: Supplementary file 1 — Supplementary Information [file 41598_2017_3780_MOESM1_ESM.pdf]

Image based Machine Learning for identification of macrophage subsets

Hassan M Rostam[1,† ¶], Paul M Reynolds[2†], Morgan R Alexander[3], Nikolaj Gadegaard[2*], Amir M Ghaemmaghami[1*]

[1]Division of Immunology, School of Life Sciences, Faculty of Medicine and Health Sciences, University of Nottingham, Nottingham, NG7 2RD, UK.

[2] Division of Biomedical Engineering, School of Engineering, University of Glasgow, Glasgow, G12 8LT. UK.

[3] Advanced Materials and Healthcare Technologies Division, School of Pharmacy, University of Nottingham, Nottingham, NG7 2RD, UK.

* Corresponding authors

† Contributed equally to the work.

¶ Current address: Department of Biology, University of Garmian, Kalar, Kurdistan

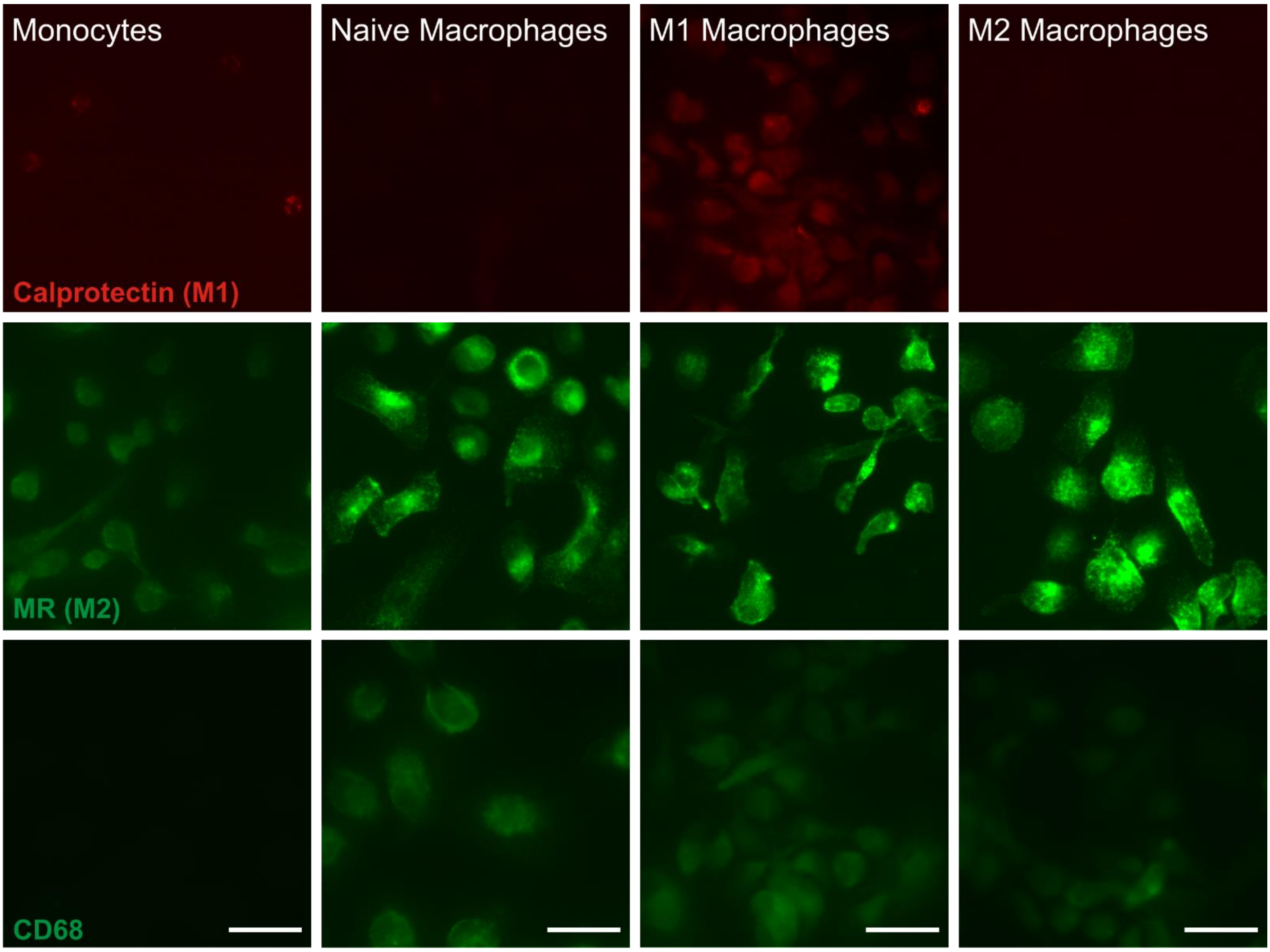

Supplementary Figure 1 Fluorescent images of cells stained for calprotectin (27E10 antigen, red), mannose receptor (MR, green), and CD68.

Scale bar = 25 µm. Representative images from n=3 are shown.

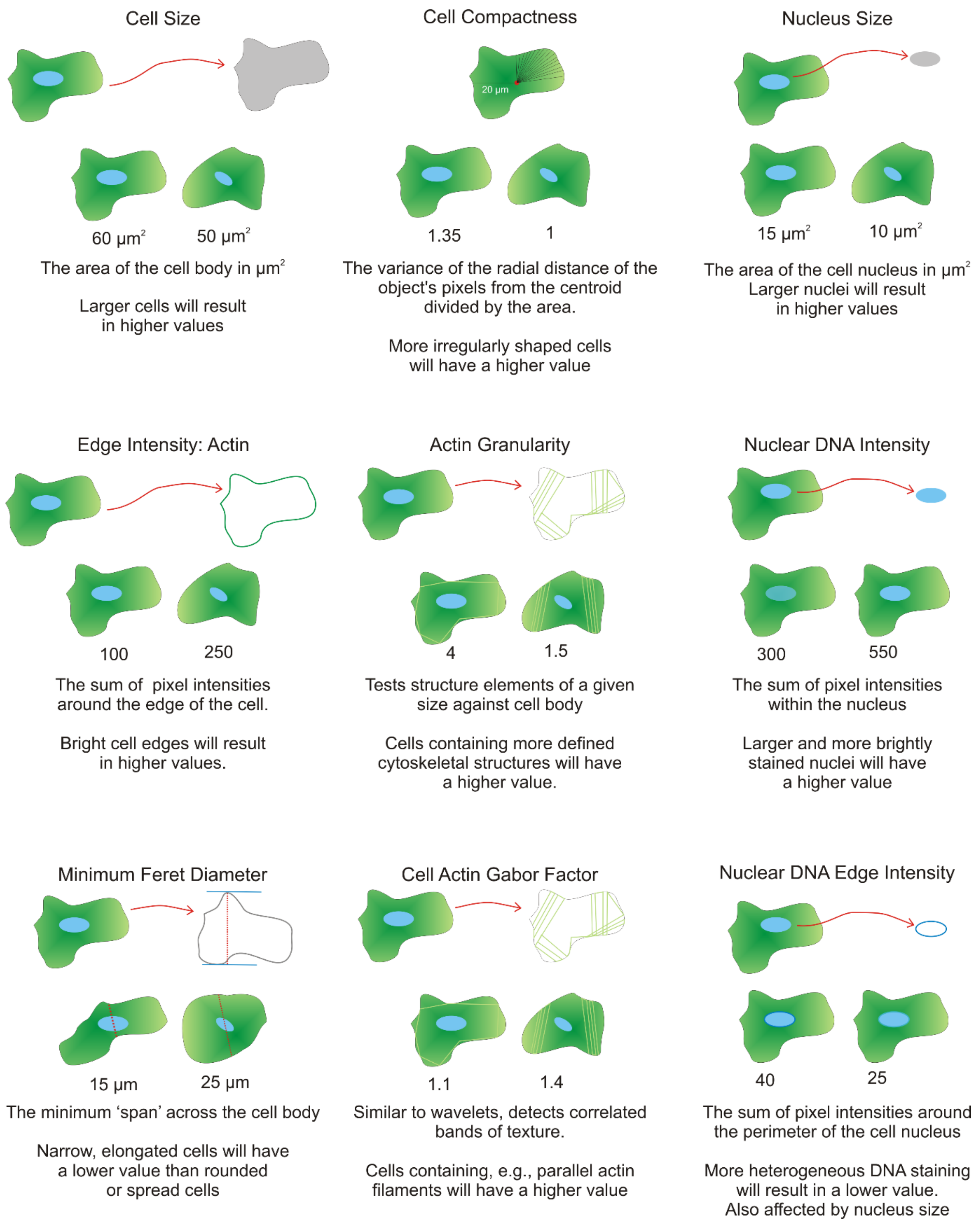

Supplementary Figure 2 Measurements used to form the cytoprofile, as plotted in figure 5 of the main text.

Logistic Regression

	M1 Macrophage	M2 Macrophage	Naïve Macrophage	Monocyte D0	Monocyte D6	∑
M1 Macrophage	98.10%	1.40%	0.00%	0.40%	0.00%	**1560**
M2 Macrophage	2.40%	94.90%	0.00%	1.50%	1.30%	**1010**
Naïve Macrophage	0.00%	0.00%	96.60%	0.10%	3.20%	**1940**
Monocyte D0	0.00%	0.50%	0.00%	95.30%	4.20%	**660**
Monocyte D6	0.00%	3.30%	11.20%	2.70%	82.80%	**950**
∑	**1555**	**1014**	**1981**	**679**	**891**	**6120**

AUC	0.963
CA	0.944
F1	0.944
Precision	0.944
Recall	0.944

Random Forest

	M1 Macrophage	M2 Macrophage	Naïve Macrophage	Monocyte D0	Monocyte D6	∑
M1 Macrophage	92.60%	1.70%	1.60%	2.10%	2.10%	**1560**
M2 Macrophage	7.00%	85.30%	5.80%	0.80%	1.00%	**1010**
Naïve Macrophage	0.90%	0.40%	96.80%	0.20%	1.80%	**1940**
Monocyte D0	7.60%	0.50%	2.10%	89.20%	0.60%	**660**
Monocyte D6	8.50%	5.10%	16.30%	2.20%	67.90%	**950**
∑	**1663**	**947**	**2130**	**654**	**726**	**6120**

AUC	0.921
CA	0.885
F1	0.882
Precision	0.886
Recall	0.885

Naïve Bayes

	M1 Macrophage	M2 Macrophage	Naïve Macrophage	Monocyte D0	Monocyte D6	∑
M1 Macrophage	78.40%	4.00%	3.10%	7.00%	7.50%	**1560**
M2 Macrophage	10.50%	78.50%	4.50%	4.90%	1.70%	**1010**
Naïve Macrophage	3.90%	7.30%	64.60%	4.30%	19.90%	**1940**
Monocyte D0	5.00%	0.30%	0.80%	93.30%	0.60%	**660**
Monocyte D6	6.50%	6.60%	13.10%	4.40%	69.40%	**950**
∑	**1500**	**1061**	**1477**	**899**	**1183**	**6120**

AUC	0.843
CA	0.743
F1	0.744
Precision	0.761
Recall	0.743

SVM

	M1 Macrophage	M2 Macrophage	Naïve Macrophage	Monocyte D0	Monocyte D6	∑
M1 Macrophage	64.10%	13.50%	6.00%	6.90%	9.50%	**1560**
M2 Macrophage	7.40%	75.60%	10.40%	0.40%	6.10%	**1010**
Naïve Macrophage	3.50%	13.60%	63.50%	1.30%	18.10%	**1940**
Monocyte D0	2.60%	2.00%	1.80%	92.10%	1.50%	**660**
Monocyte D6	7.80%	10.30%	16.50%	2.60%	62.70%	**950**
∑	**1234**	**1349**	**1600**	**770**	**1167**	**6120**

AUC	0.806
CA	0.686
F1	0.689
Precision	0.709
Recall	0.686

kNN

	M1 Macrophage	M2 Macrophage	Naïve Macrophage	Monocyte D0	Monocyte D6	∑
M1 Macrophage	64.80%	3.80%	15.50%	9.40%	6.50%	**1560**
M2 Macrophage	23.60%	44.50%	24.30%	4.70%	3.10%	**1010**
Naïve Macrophage	27.40%	10.20%	48.50%	4.80%	9.00%	**1940**
Monocyte D0	14.70%	1.70%	7.40%	75.50%	0.80%	**660**
Monocyte D6	25.10%	11.10%	41.30%	3.80%	18.80%	**950**
∑	**2116**	**822**	**1869**	**821**	**492**	**6120**

AUC	0.674
CA	0.503
F1	0.489
Precision	0.493
Recall	0.503

Supplementary Table 1 Comparison of 5 classifier models used to segment immunofluorescent data. Confusion matrices are presented for each classifier, alongside accuracy metrics AUC (area under curve), CA (classifier accuracy), F1 (F score), Precision and Recall. All metrics are a result of 10 fold cross validation analysis.

Algorithm	Parameter settings
Logistic regression	**Regularization:** Ridge (L2), C=1
Random forest	**Number of trees:** 20<br>**Maximal number of considered features:** 5<br>**Maximal tree depth:** unlimited<br>**Stop splitting nodes with maximum instances:** 5
Naïve Bayes	-
SVM	**SVM type:** C-SVM, C=1.0<br>**Kernel:** RBF, $\exp(-1.0\|x-y\|^2)$<br>Numerical tolerance: 0.001<br>**Iteration limit:** 100
kNN	**Number of neighbours:** 10<br>**Metric:** Euclidean<br>**Weight:** Uniform

Supplementary Table 2 Details of classifiers used for segmentation of immune cells
